# Size-dependent heterogeneity in the associations of C-TIRADS ultrasound features with malignancy in thyroid nodules

**DOI:** 10.3389/fendo.2026.1844286

**Published:** 2026-10-07

**Authors:** Dongming Guo, Yonghua Xu, Xianying Liao, Haiqing Huang, Yuxia Zhai, Haitao Li, Zhihui Lin, Jiajia Wang, Zhe Chen

**Affiliations:** 1 Department of Interventional Ultrasound, Cancer Hospital of Shantou University Medical College, Shantou, China; 2 Department of Interventional Ultrasound, Second Affiliated Hospital of Shantou University Medical College, Shantou, China; 3 Department of Ultrasound, Cancer Hospital of Shantou University Medical College, Shantou, China; 4 Department of Ultrasound, Second Affiliated Hospital of Shantou University Medical College, Shantou, China; 5 Department of Thoracic Surgery, First Affiliated Hospital of Shantou University Medical College, Shantou, China

**Keywords:** C-TIRADS, heterogeneity, malignancy risk, nodule size, thyroid nodules, ultrasonography

## Abstract

**Aim:**

To investigate whether the associations between Chinese Thyroid Imaging Reporting and Data System (C-TIRADS) ultrasound features and malignancy vary according to thyroid nodule size.

**Methods:**

This two-center retrospective cohort study included 3726 thyroid nodules, which were categorized according to maximum diameter as ≤1 cm or >1 cm. Extreme gradient boosting models and SHapley Additive exPlanations (SHAP) were used for exploratory visualization of the contributions of individual ultrasound features to malignancy prediction in the overall cohort and the two size subgroups. Multivariable Firth logistic regression was used to estimate the adjusted associations between the C-TIRADS features and malignancy. Single-feature interaction models were constructed to assess whether nodule size modified these associations. The discriminative performance of the original C-TIRADS model and the interaction model was compared using the area under the receiver operating characteristic curve (AUC). Center-adjusted sensitivity analyses were also performed.

**Results:**

Vertical orientation and solid composition showed greater contributions to malignancy prediction and stronger associations with malignancy in nodules ≤1 cm, whereas microcalcification and ill-defined/irregular margin or extrathyroidal extension showed stronger associations with malignancy in nodules >1 cm. Significant feature-by-size interactions were identified for vertical orientation, solid composition, microcalcification, and ill-defined/irregular margin or extrathyroidal extension, whereas no significant interactions were observed for markedly hypoechoic or comet-tail artifact. The interaction model showed a modest but statistically significant improvement in discriminative performance compared with the original C-TIRADS model (AUC 0.918 vs. 0.911, *P* < 0.001). The center-adjusted sensitivity analyses yielded consistent findings.

**Conclusion:**

The associations between C-TIRADS ultrasound features and malignancy exhibit significant size-dependent heterogeneity. In nodules ≤1 cm, vertical orientation and solid composition may indicate a substantially higher likelihood of malignancy and therefore warrant particularly careful clinical evaluation, whereas microcalcification and ill-defined/irregular margin or extrathyroidal extension may be more informative for malignancy assessment in nodules >1 cm. Considering nodule size when interpreting individual C-TIRADS features may improve diagnostic discrimination.

## 1 Introduction

Thyroid nodules are highly prevalent in the general population and rank among the most frequent disorders of the endocrine system. Nodule size is related to malignancy risk and may influence diagnostic performance in some way (1–3). However, most existing risk-stratification systems implicitly assume that the diagnostic contribution of each ultrasound feature remains broadly stable across nodules of different sizes (4–6). In practice, nodule size is mainly used to guide biopsy thresholds or follow-up intervals rather than to modify the interpretation of individual suspicious features (7). This assumption may be overly simplistic. Nodules with maximum diameter ≤1 cm often present a distinct diagnostic challenge (8). Their small size may limit the conspicuity of some classic malignant signs. Smaller nodules (<10 mm) tend to show lower stiffness parameters (9) and rarely demonstrate capsular invasion or extrathyroidal extension (10). Certain morphologic features may become more informative at an earlier stage of tumor development. Vertical orientation is more common in small nodules at an early stage, with its incidence declining linearly as nodules grow larger in volume (11). Moreover, accurate identification of early small nodules is impeded by inherent speckle noise and complex anatomical backgrounds in sonographic images (12). Previous studies (13, 14) have reported size-dependent differences in the performance of ultrasound risk-stratification systems and individual sonographic features. Xu et al. found that the diagnostic performance of the 2015 American Thyroid Association ultrasound patterns varied across size groups and that the system was less reliable in nodules <10 mm (13). Lyshchik et al. reported that the most reliable diagnostic criteria for malignancy were an irregular outline, subcapsular location, and increased intranodular vascularity in nodules ≤15 mm, whereas hypoechogenicity served as the only reliable malignant sign in nodules >15 mm (14). Thus, the value of suspicious sonographic features may not be constant across different nodule sizes.

The 1-cm cutoff is one of the most commonly used size thresholds to guide decisions regarding fine-needle aspiration and surveillance in the evaluation and management of thyroid nodules. Several major thyroid ultrasound risk-stratification guidelines, including the American College of Radiology Thyroid Imaging Reporting and Data System (4), the European Thyroid Association Guidelines for Ultrasound Malignancy Risk Stratification of Thyroid Nodules (5), and the Korean Thyroid Imaging Reporting and Data System (6), incorporate approximately 1 cm as an important threshold for fine-needle aspiration or further evaluation of highly suspicious nodules. Moreover, papillary thyroid carcinomas measuring ≤1 cm are conventionally classified as papillary thyroid microcarcinomas, for which management considerations may differ from those for larger tumors, including the option of active surveillance in appropriately selected low-risk patients (15–17). Therefore, the 1-cm cutoff represents a clinically meaningful boundary rather than an arbitrary statistical division. Given that biopsy and management decisions for thyroid nodules vary according to the 1-cm threshold, it is clinically relevant to investigate whether the associations between ultrasound features and malignancy differ between nodules ≤1 cm and those >1 cm.

The Chinese Thyroid Imaging Reporting and Data System (C-TIRADS) is one of the most widely used thyroid ultrasound risk-stratification systems in China (18). Although its overall diagnostic performance has been investigated in several studies (19–21), the size-dependent heterogeneity in the associations between C-TIRADS ultrasound features and malignancy risk has not been sufficiently clarified (22). In addition, most prior studies (8, 23, 24) have concentrated on the diagnosis and clinical management of thyroid nodules ≤1 cm. However, it has not been thoroughly explored whether a given ultrasound feature yields equal diagnostic efficacy in nodules ≤1 cm and those exceeding 1 cm. A clearer understanding of this issue is important, because it may refine how clinicians interpret established suspicious signs in small versus larger nodules, even if the current scoring systems do not emphasize that.

Therefore, the aim of the present study was to investigate whether the associations of C-TIRADS ultrasound features with malignancy risk differ between thyroid nodules ≤1 cm and those >1 cm.

## 2 Methods

### 2.1 Study design and setting

This multicenter retrospective cohort study was conducted at the Second Affiliated Hospital of Shantou University Medical College and Cancer Hospital of Shantou University Medical College. Consecutive patients evaluated at the first institution between January 2019 and May 2025 and at the second institution between June 2020 and May 2025 were retrospectively screened for eligibility.

### 2.2 Sample size, sampling method, and eligibility criteria

Consecutive sampling was performed, and all potentially eligible patients during the study period were initially screened. Patients were initially eligible if they underwent thyroid ultrasonography at either participating institution and subsequently underwent ultrasound-guided biopsy or surgical resection. For patients with multiple nodules, the nodule with the highest C-TIRADS category was selected. If two or more nodules had the same category, the largest nodule was included. The final diagnosis was strictly mandated to be substantiated by either cytology or postoperative pathology.

After initial screening, nodules were further excluded for the following reasons: (1) incomplete imaging or clinical information; (2) poor-quality ultrasound imaging that precluded accurate nodule assessment; (3) ambiguous diagnostic outcomes that lacked a definitive benign or malignant classification; and (4) a history of neck irradiation or oncological therapy before the current thyroid diagnosis. Nodules were divided into two groups according to nodule size: nodules ≤1 cm and nodules >1 cm.

### 2.3 Data collection

Demographic and clinical data, including age, sex, and cytologic or histopathologic diagnoses, were retrospectively extracted from the electronic medical records. Nodule maximum diameter and the six C-TIRADS ultrasound features were obtained from ultrasound reports and archived images within the ultrasound imaging system.

Ultrasound examinations were performed with patients in the supine position and the neck mildly extended. At Cancer Hospital of Shantou University Medical College, examinations were performed mainly using Resona 7 (Mindray, Shenzhen, China) and EPIQ 7 (Philips Ultrasound, Bothell, WA, USA) ultrasound systems. At the Second Affiliated Hospital of Shantou University Medical College, examinations were performed mainly using Resona 7 (Mindray, Shenzhen, China), EPIQ 7 (Philips Ultrasound, Bothell, WA, USA), LOGIQ E9 (GE Healthcare, Wauwatosa, WI, USA), and RS80A (Samsung Medison, Seoul, Republic of Korea) ultrasound systems.

Each thyroid nodule was systematically examined in both transverse and longitudinal planes. The size of the nodule was recorded, and the following six ultrasound features were assessed according to the 2020 C-TIRADS criteria (18): (1) solid; (2) markedly hypoechoic; (3) vertical orientation; (4) microcalcification; (5) ill-defined/irregular margin or extrathyroidal extension; and (6) comet-tail artifact.

Ultrasound features were retrospectively extracted from the original ultrasound reports and independently verified using the archived images by two ultrasound physicians (DMG and ZC), each with more than 5 years of experience in thyroid ultrasonography. The reviewers were blinded to the cytologic or histopathologic diagnosis at the time of image interpretation. Any disagreements were resolved by a third senior ultrasound physician (YXZ) who had more than 20 years of experience in thyroid ultrasonography.

### 2.4 XGBoost and SHAP analysis

To provide a model-based visualization of feature contribution patterns across size groups, extreme gradient boosting (XGBoost) models were established separately in the overall cohort, the ≤1 cm subgroup, and the >1 cm subgroup using the six C-TIRADS features as predictors. For each model, the values of SHapley Additive exPlanations (SHAP) were calculated, and the mean absolute SHAP value was used to quantify the overall contribution of each feature to model prediction. SHAP bar plots were generated to compare feature-importance patterns across the three groups.

### 2.5 Evaluation of independent risk

Due to the asymmetrical distribution of the comet-tail artifact, which was predominantly observed in benign lesions and remained exceedingly rare in malignant cases, conventional logistic regression was prone to sparse-data bias or quasi-complete separation. Consequently, Firth penalized logistic regression was implemented to provide more stable and unbiased parameter estimates for the association analyses. Multivariable Firth logistic regression models with odds ratios (ORs) and 95% confidence intervals (CIs) were fitted in the overall cohort and separately in the ≤1 cm and >1 cm subgroups to evaluate the independent association of each feature with malignancy across the three groups.

### 2.6 Single-feature interaction analysis

To assess whether the association between each ultrasound feature and malignancy differed according to nodule size, single-feature interaction analyses were performed using multivariable Firth logistic regression. Each interaction model included the six C-TIRADS ultrasound features, nodule size group, and one feature-by-size interaction term. In the interaction models, malignancy was specified as the outcome variable. For each ultrasound feature, the OR represented the adjusted odds ratio for malignancy associated with the presence versus absence of that feature among nodules >1 cm, which served as the reference group. The interaction OR represented the ratio of the OR for that feature in nodules ≤1 cm to the corresponding OR in nodules >1 cm, thereby quantifying how nodule size modified the association between the ultrasound feature and malignancy.

### 2.7 Model comparison

To examine the overall incremental value of incorporating size-dependent interactions, the following two multivariable Firth logistic regression models were compared: (1) the original C-TIRADS model including the six ultrasound features; (2) an interaction model additionally including the nodule size category and the significant feature-by-size interaction terms. The discriminative performance of the two models was evaluated using receiver operating characteristic (ROC) curves and the area under the ROC curve (AUC). DeLong’s test was used to compare correlated ROC curves.

### 2.8 Sensitivity analysis

To evaluate whether the observed size-dependent heterogeneity was driven by inter-center differences, sensitivity analyses were performed by additionally adjusting for study center in the single-feature interaction models. The direction, magnitude, and statistical significance of the interaction terms were compared with those of the primary analysis.

### 2.9 Statistical analysis

Continuous variables were presented as mean ± standard deviation or median with interquartile range. Categorical variables were described as counts and percentages. Between-group comparisons were performed using Student’s t test or Mann–Whitney U test for continuous variables and the chi-square test or Fisher’s exact test for categorical variables, as appropriate.

All statistical analyses were performed using R (version 4.5.3). The construction of XGBoost models and calculation of SHAP values were conducted using the xgboost package (version 3.2.0.1). A two-sided *P*-value <0.05 was considered statistically significant.

## 3 Results

### 3.1 Patient selection

A total of 4077 potentially eligible patients were screened at the two participating institutions. After exclusion of 351 patients according to the predefined eligibility criteria, 3726 patients with 3726 thyroid nodules were included in the final analysis. Of these, 1214 nodules measured ≤1 cm and 2512 measured >1 cm. The detailed process of patient-selection is shown in **Figure 1**.

**Figure 1:**
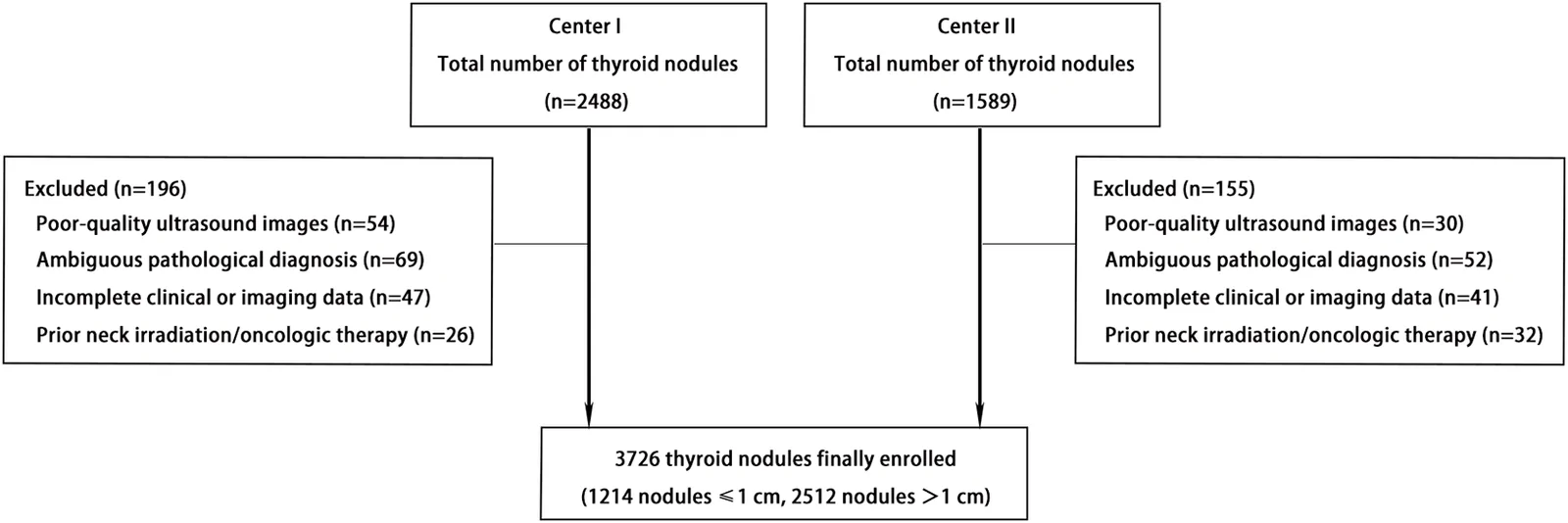
Study flowchart of nodule selection.

### 3.2 Baseline characteristics

The baseline characteristics of the included nodules are summarized in **Table 1**. Compared with nodules >1 cm, nodules ≤1 cm showed a higher prevalence of malignancy and higher frequencies of vertical orientation, solid composition, markedly hypoechoic, microcalcification, and ill-defined/irregular margin or extrathyroidal extension, whereas comet-tail artifact was less frequent (all *P* < 0.001). Patients with nodules ≤1 cm were slightly older (*P* = 0.003), while the sex distribution was similar between the two groups (*P* = 0.279).

**Table 1:** Baseline characteristics of thyroid nodules according to nodule size.

Variable	Nodules ≤1 cm (n=1214)	Nodules >1 cm (n=2512)	*P* value
Age, years	48 (37-57)	46 (33-57)	0.003
Female, n (%)	1010 (83.2)	2052 (81.7)	0.279
Malignancy, n (%)	610 (50.2)	659 (26.2)	<0.001
Vertical orientation, n (%)	480 (39.5)	189 (7.5)	<0.001
Solid, n (%)	1017 (83.8)	1514 (60.3)	<0.001
Markedly hypoechoic, n (%)	80 (6.6)	75 (3.0)	<0.001
Microcalcification, n (%)	363 (29.9)	540 (21.5)	<0.001
Ill-defined/irregular margin or extrathyroidal extension, n (%)	392 (32.3)	551 (21.9)	<0.001
Comet-tail artifact, n (%)	35 (2.9)	176 (7.0)	<0.001

### 3.3 SHAP feature importance across size groups

In the overall cohort, the most important features according to mean absolute SHAP values were microcalcification (0.814), vertical orientation (0.789), and ill-defined/irregular margin or extrathyroidal extension (0.751), followed by solid (0.540), comet-tail artifact (0.155), and markedly hypoechoic (0.099).

In nodules ≤1 cm, vertical orientation showed the highest contribution to the prediction of malignancy (1.879), followed by solid (0.841), ill-defined/irregular margin or extrathyroidal extension (0.538), and microcalcification (0.503). In contrast, in nodules >1 cm, microcalcification had the highest contribution to the prediction of malignancy (0.920), followed by ill-defined/irregular margin or extrathyroidal extension (0.785), vertical orientation (0.605), and solid (0.484).

Overall, SHAP analysis suggested that vertical orientation and solid contributed more strongly to malignancy prediction in nodules ≤1 cm, whereas microcalcification and ill-defined/irregular margin or extrathyroidal extension emerged as more substantial contributors in nodules >1 cm (**Figure 2**).

**Figure 2:**
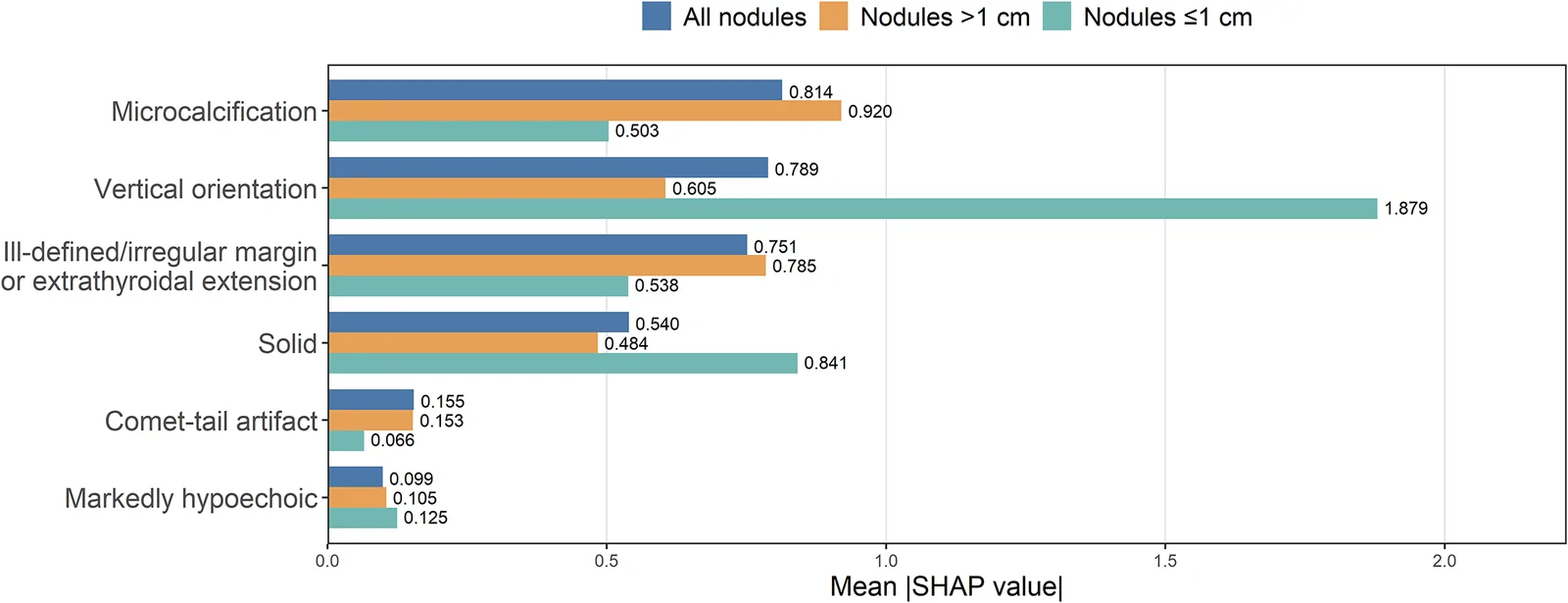
SHAP-based feature importance of C-TIRADS ultrasound features across nodule-size groups.

### 3.4 Multivariable Firth logistic regression across size groups

In the overall cohort, vertical orientation showed the strongest association with malignancy (OR 14.76, 95% CI 11.14-19.74), followed by microcalcification (OR 8.08, 95% CI 6.47-10.13), ill-defined/irregular margin or extrathyroidal extension (OR 6.54, 95% CI 5.24-8.20), markedly hypoechoic (OR 3.99, 95% CI 2.37-6.86), and solid (OR 3.41, 95% CI 2.62-4.48). Comet-tail artifact was inversely associated with malignancy (OR 0.10, 95% CI 0.02-0.31).

In nodules ≤1 cm, vertical orientation (OR 18.95, 95% CI 13.06-28.05) and solid (OR 7.21, 95% CI 3.77-15.10) were the most prominent independent risk features for malignancy, followed by ill-defined/irregular margin or extrathyroidal extension (OR 4.28, 95% CI 2.95-6.27), markedly hypoechoic (OR 3.57, 95% CI 1.60-8.78), and microcalcification (OR 3.26, 95% CI 2.23-4.80). Comet-tail artifact remained significantly associated with benignity (OR 0.06, 95% CI 0.0004-0.55).

In nodules >1 cm, microcalcification (OR 12.78, 95% CI 9.69-16.94) and ill-defined/irregular margin or extrathyroidal extension (OR 7.93, 95% CI 6.00-10.52) were the strongest malignant features, followed by vertical orientation (OR 5.51, 95% CI 3.42-9.00), markedly hypoechoic (OR 5.16, 95% CI 2.65-10.14), and solid (OR 2.83, 95% CI 2.08-3.87). Comet-tail artifact again showed an inverse association with malignancy (OR 0.17, 95% CI 0.03-0.50).

Taken together, these subgroup analyses indicated that vertical orientation and solid exhibited a stronger association with malignancy in nodules ≤1 cm, whereas microcalcification and ill-defined/irregular margin or extrathyroidal extension were identified as more prominent risk factors for malignancy in nodules >1 cm (**Figure 3**).

**Figure 3:**
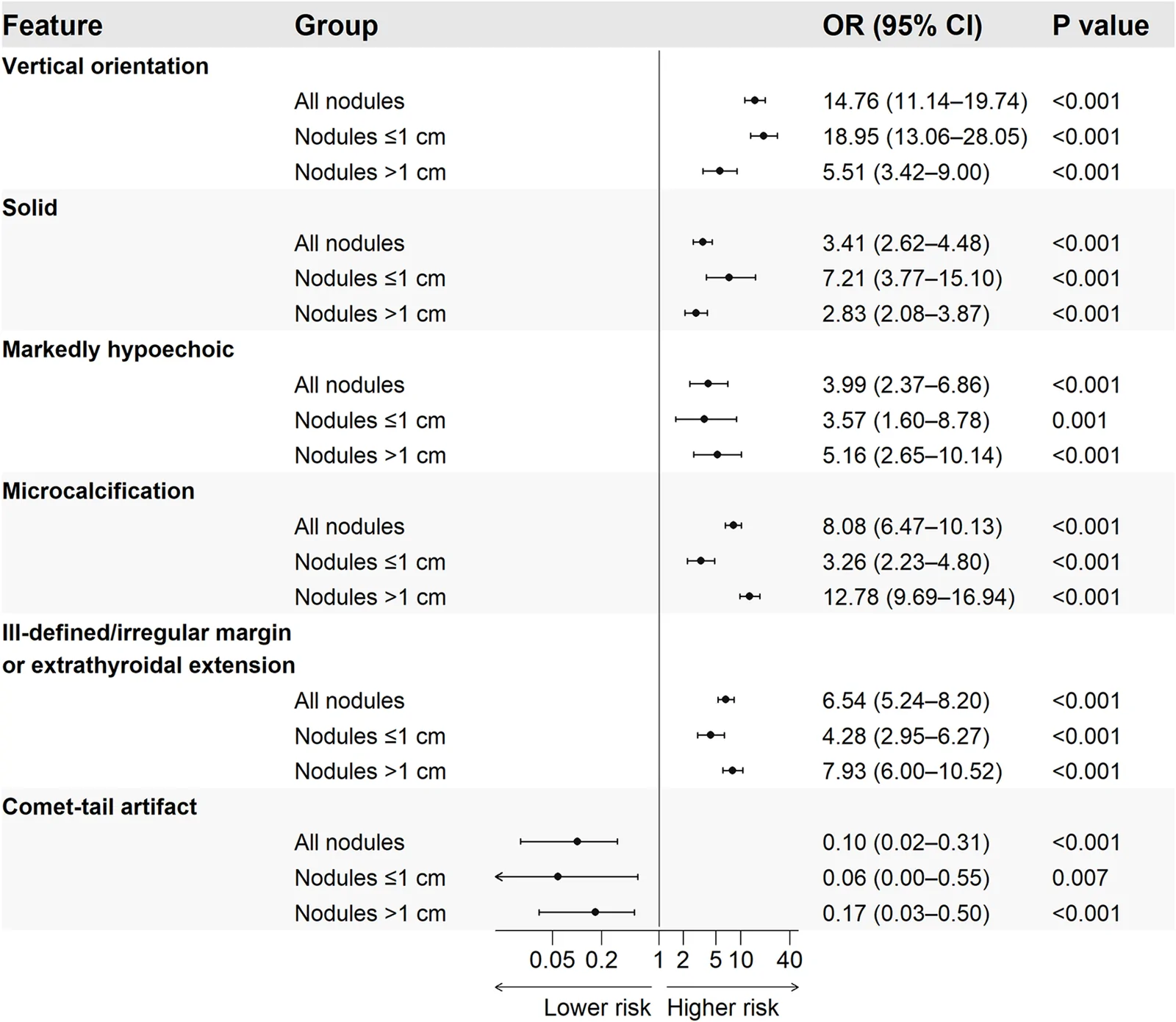
Forest plot of multivariable Firth logistic regression results across nodule-size groups.

### 3.5 Single-feature interaction analysis

The interaction results are summarized in **Table 2**. Significant interactions with size group were observed for vertical orientation, solid, microcalcification, and ill-defined/irregular margin or extrathyroidal extension.

**Table 2:** Single-feature interaction analyses between C-TIRADS ultrasound features and nodule size group using multivariable Firth logistic regression.

Features	OR in >1 cm (95% CI)	Interaction OR (95% CI)	*P* for interaction
Vertical orientation	5.24 (3.32-8.39)	4.54 (2.47-8.32)	<0.001
Solid	2.79 (2.08-3.78)	2.43 (1.20-5.32)	0.013
Markedly hypoechoic	4.30 (2.22-8.40)	0.80 (0.28-2.46)	0.687
Microcalcification	12.97 (9.84-17.18)	0.24 (0.15-0.39)	<0.001
Ill-defined/irregular margin or extrathyroidal extension	7.97 (6.06-10.53)	0.56 (0.35-0.89)	0.014
Comet-tail artifact	0.14 (0.03-0.42)	0.57 (0.004-8.46)	0.720

The OR in nodules >1 cm represents the association between the presence of each feature and malignancy in the nodules >1 cm group. The interaction OR is the ratio of the corresponding OR in nodules ≤1 cm to that in nodules >1 cm; values >1 indicate a stronger association in nodules ≤1 cm, whereas values <1 indicate a stronger association in nodules >1 cm.

C-TIRADS, Chinese Thyroid Imaging Reporting and Data System; OR, odds ratio; CI, confidence interval.

For vertical orientation, the adjusted OR for malignancy in nodules >1 cm was 5.24 (95% CI 3.32-8.39), and the interaction OR was 4.54 (95% CI 2.47-8.32; *P* < 0.001), indicating that its association with malignancy was substantially stronger in nodules ≤1 cm. For solid composition, the adjusted OR for malignancy in nodules >1 cm was 2.79 (95% CI 2.08-3.78), and the interaction OR was 2.43 (95% CI 1.20-5.32; *P* = 0.013), likewise indicating a stronger association with malignancy in nodules ≤1 cm. In contrast, microcalcification showed a stronger association with malignancy in nodules >1 cm. Its adjusted OR for malignancy in nodules >1 cm was 12.97 (95% CI 9.84-17.18), whereas the interaction OR was 0.24 (95% CI 0.15-0.39; *P* < 0.001), indicating a weaker association in nodules ≤1 cm. Similarly, ill-defined/irregular margin or extrathyroidal extension had an adjusted OR for malignancy of 7.97 (95% CI 6.06-10.53) in nodules >1 cm, with an interaction OR of 0.56 (95% CI 0.35-0.89; *P* = 0.014), also indicating a weaker association in nodules ≤1 cm.

No significant interaction with size was observed for markedly hypoechoic (interaction OR 0.80, 95% CI 0.28-2.46; *P* = 0.687) or comet-tail artifact (interaction OR 0.57, 95% CI 0.004-8.46; *P* = 0.720).

### 3.6 Comparison of the original and interaction models

As shown in **Figure 4**, the interaction model showed a modest but statistically significant improvement in diagnostic efficacy compared with the original C-TIRADS model. The AUC increased from 0.911 for the original model to 0.918 for the interaction model (*P* < 0.001).

**Figure 4:**
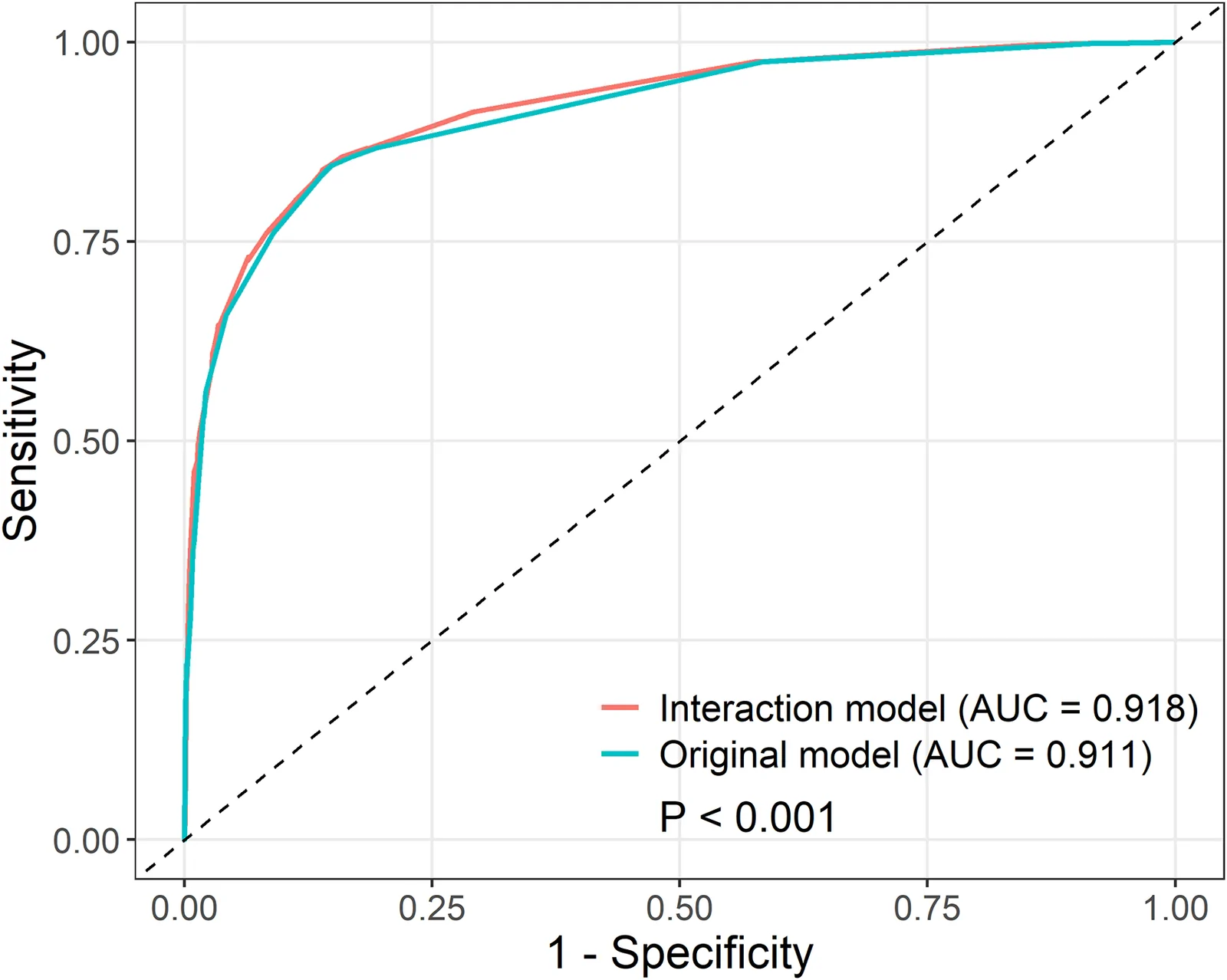
Receiver operating characteristic curves of the original and interaction models.

### 3.7 Center-adjusted sensitivity analysis

Sensitivity analyses demonstrated that the major interaction findings remained directionally and statistically consistent with the primary analysis (**Table 3**). Specifically, the interaction terms for vertical orientation (interaction OR 4.55, 95% CI 2.49-8.33; *P* < 0.001), solid (interaction OR 2.46, 95% CI 1.21-5.39; *P* = 0.011), microcalcification (interaction OR 0.25, 95% CI 0.15-0.39; *P* < 0.001), and ill-defined/irregular margin or extrathyroidal extension (interaction OR 0.55, 95% CI 0.35-0.89; *P* = 0.014) all remained significant after adjustment for center.

**Table 3:** Sensitivity analysis of single-feature interaction models after adjustment for study center using multivariable Firth logistic regression.

Features	OR in >1 cm (95% CI)	Interaction OR (95% CI)	*P* for interaction
Vertical orientation	5.19 (3.30-8.30)	4.55 (2.49-8.33)	<0.001
Solid	2.80 (2.09-3.79)	2.46 (1.21-5.39)	0.011
Markedly hypoechoic	4.17 (2.16-8.14)	0.86 (0.30-2.64)	0.779
Microcalcification	12.96 (9.83-17.16)	0.25 (0.15-0.39)	<0.001
Ill-defined/irregular margin or extrathyroidal extension	7.82 (5.94-10.34)	0.55 (0.35-0.89)	0.014
Comet-tail artifact	0.14 (0.03-0.43)	0.58 (0.004-8.57)	0.728

The OR in nodules >1 cm represents the adjusted association between the presence of each feature and malignancy in the nodules >1 cm group. The interaction OR is the ratio of the corresponding adjusted OR in nodules ≤1 cm to that in nodules >1 cm; values >1 indicate a stronger association in nodules ≤1 cm, whereas values <1 indicate a stronger association in nodules >1 cm.

C-TIRADS, Chinese Thyroid Imaging Reporting and Data System; OR, odds ratio; CI, confidence interval.

By contrast, markedly hypoechoic (interaction OR 0.86, 95% CI 0.30-2.64; *P* = 0.779) and comet-tail artifact (interaction OR 0.58, 95% CI 0.004-8.57; *P* = 0.728) remained non-significant. These findings suggest that the observed size-dependent heterogeneity of C-TIRADS features was not solely attributable to inter-center differences.

## 4 Discussion

In this multicenter study, we found that the associations between C-TIRADS ultrasound features and malignancy were not uniform across thyroid nodules of different sizes. SHAP analysis suggested distinct feature-importance patterns among all nodules, nodules ≤1 cm, and nodules >1 cm, and these differences were subsequently confirmed by multivariable Firth regression and formal interaction analyses. Specifically, vertical orientation and solid carried greater weight in identifying malignancy in nodules ≤1 cm, whereas microcalcification and ill-defined/irregular margin or extrathyroidal extension were more important for nodules >1 cm. Incorporation of these size-dependent interactions resulted in a modest but statistically significant improvement in overall diagnostic efficacy, indicating that nodule size modifies the diagnostic value of several established sonographic features rather than serving only as a background descriptive variable.

One of the most striking findings of this study was that vertical orientation exhibited the strongest association with malignancy and played a more prominent role in nodules ≤1 cm compared with nodules >1 cm, which was in line with the earlier reports (10, 22, 25). The taller-than-wide sign reflects growth across normal tissue planes and has long been considered one of the more specific ultrasound indicators of malignancy (26). Prior research (25) has also emphasized the importance of this sign, especially when combined with microcalcification. One possible explanation for its stronger effect in subcentimeter nodules is that small malignant lesions grow infiltratively across tissue planes, whereas larger nodules may eventually be constrained by the thyroid capsule or surrounding anatomical structures, leading to a loss of the taller-than-wide characteristic. Thus, in small nodules, shape-related distortion may provide particularly important diagnostic information at an early stage of malignant growth.

Solid also showed a stronger association with malignancy in nodules ≤1 cm. This finding suggests that structural morphology may be especially informative in small nodules. Small malignant nodules often present as compact, fully solid lesions without a mixed architecture. In the C-TIRADS, K-TIRADS, and ACR-TIRADS lexicons (4, 6, 18), solid composition is integrated into malignancy risk assessment, but its diagnostic contribution is not explicitly modeled as size-dependent. Our results suggest that the contribution of solidity may be amplified in the subcentimeter setting, particularly when interpreted together with vertical orientation. This may help explain why some small nodules with otherwise limited suspicious findings still show a high probability of malignancy when they are both solid and taller-than-wide.

In contrast, microcalcification and ill-defined/irregular margin or extrathyroidal extension were more strongly associated with malignancy in nodules >1 cm. This result was highly consistent across SHAP, stratified Firth regression, and interaction analysis. Microcalcification and irregular margin have long been regarded as hallmark suspicious findings of malignant nodules (27). Earlier work reported that approximately half of papillary thyroid microcarcinomas showed microcalcifications and ill-defined margins (28), supporting their relevance even in very small cancers. However, our data indicate that although microcalcification and ill-defined/irregular margin or extrathyroidal extension remain associated with malignancy in both groups, their relative discriminatory contribution becomes greater once nodules exceed 1 cm. Similarly, previous research reported that the sensitivity of microcalcification in subcentimeter nodules was lower than that in larger nodules (29). Larger malignant nodules often exhibit more aggressive growth rates. As the tumor expands rapidly, central areas may outgrow their blood supply, leading to ischemic necrosis. The subsequent deposition of calcium salts in these necrotic foci results in the formation of psammoma bodies, which manifest as microcalcifications on ultrasound. Additionally, larger malignant nodules may have had more time to develop infiltrative growth patterns that become sonographically appreciable as irregular margins or evidence of extrathyroidal extension. In other words, these classic malignant signs may represent later or more fully expressed morphologic consequences of tumor progression, and therefore contribute more strongly in larger nodules.

Markedly hypoechoic behaved differently. Although it was independently associated with malignancy in the three groups, its interaction with size was not statistically significant. This suggests that markedly hypoechoic is indeed a suspicious feature, but its effect may be more stable across size groups than that of vertical orientation, solid, microcalcification, or margin status. The absence of a significant interaction implies that markedly hypoechoic may function as a more general malignant feature rather than one whose diagnostic meaning changes strongly with nodule size.

Comet-tail artifact consistently behaved as a benign-associated feature, but its interaction with size was not significant. Comet-tail artifact has traditionally been linked with colloid-containing benign nodules (30, 31). Although sparse-data issues of comet-tail artifact can make modeling unstable in ordinary logistic regression, its direction of effect in our Firth models was remarkably consistent. The lack of a significant interaction suggests that comet-tail artifact retains a broadly similar benign implication across size groups, even if its absolute prevalence is low. Thus, comet-tail artifact does not appear to be a driver of size-related diagnostic heterogeneity in our study.

An important aspect of our study is that the machine-learning and regression-based results were concordant. The SHAP bar plots provided a visual, model-based summary of feature-importance patterns, while the Firth models and interaction analyses supplied formal effect estimates and statistical inference. This combination strengthens interpretability. SHAP alone can show ranking differences but cannot establish whether an observed shift in importance is statistically meaningful. Conversely, regression interaction terms can quantify heterogeneity. Thus, our study provides mutually reinforcing evidence that several C-TIRADS features do not contribute equally across all nodule sizes.

Although the increase in AUC from the original model to the interaction model was modest, it remained statistically significant. We do not view this as a contradiction to the interaction findings. In diagnostic modeling, especially when strong main effects are already present, a feature can show meaningful effect modification without producing a large gain in AUC when added as an interaction term. AUC is relatively insensitive to some forms of incremental improvement, particularly when baseline discrimination is already high. Therefore, the small but significant increase in AUC should be interpreted as supportive rather than central evidence that accounting for size-dependent heterogeneity has measurable overall value.

The sensitivity analyses further support the robustness of these findings. After adjustment for study center effect, the significant interaction terms, including vertical orientation, solid, microcalcification, and ill-defined/irregular margin or extrathyroidal extension, remained directionally and statistically consistent, whereas markedly hypoechoic and comet-tail artifact remained non-significant. This suggests that the observed heterogeneity was not simply an artifact of center-specific imaging practice or case mix.

Our study also has potential implications for how size is conceptualized in ultrasound risk stratification. Existing TIRADS frameworks are built around suspicious features whose diagnostic roles are assumed to be broadly stable, while nodule size is mainly used to guide biopsy or follow-up thresholds (32). Our results suggest that this assumption may be overly simplistic. Size appears to function not only as a management threshold but also as an effect modifier of key sonographic signs. Our findings support a more nuanced interpretation of suspicious features according to lesion size, particularly for subcentimeter nodules.

Several limitations should be acknowledged. This was a retrospective study, and selection bias is inevitable. Additionally, although the multicenter design was a strength, our findings still require confirmation in broader external populations.

## 5 Conclusions

In conclusion, the associations of individual C-TIRADS ultrasound features with malignancy exhibit significant size-dependent heterogeneity. Nodule size should be considered not only as a threshold for clinical management but also as an important context for interpreting the diagnostic significance of individual ultrasound features. Incorporating nodule size into feature-based malignancy risk assessment may improve diagnostic discrimination. Specifically, in nodules ≤1 cm, vertical orientation and solid composition may indicate a substantially higher likelihood of malignancy and therefore warrant particularly careful clinical evaluation, whereas microcalcification and ill-defined/irregular margin or extrathyroidal extension may be more informative for malignancy assessment in nodules >1 cm.

## Data Availability

The original contributions presented in the study are included in the article/supplementary material. Further inquiries can be directed to the corresponding author.
